# Epidemiological Investigation of the 2019 Dengue Outbreak in Dhaka, Bangladesh

**DOI:** 10.1155/2023/8898453

**Published:** 2023-03-16

**Authors:** Sabrina Yesmin, Shahnoor Sarmin, Alamgir Mustak Ahammad, Md. Abdur Rafi, Mohammad Jahid Hasan

**Affiliations:** ^1^BIRDEM General Hospital, Dhaka, Bangladesh; ^2^Dhaka Medical College, Dhaka, Bangladesh; ^3^Sherpur District Hospital, Sherpur, Bangladesh; ^4^Pi Research Consultancy Center, Dhaka, Bangladesh; ^5^Tropical Disease and Health Research Center, Dhaka, Bangladesh

## Abstract

**Introduction:**

Bangladesh experienced its largest dengue epidemic in 2019. Our objective was to investigate the epidemiological, clinical, and laboratory characteristics of the hospital-admitted dengue patients during this epidemic.

**Methods:**

This cross-sectional study was conducted among 369 adult dengue patients admitted to two tertiary care hospitals in Dhaka, Bangladesh, from June to September 2019. The disease severity was determined according to the WHO's 2009 classification.

**Results:**

The average age of the patients was 33.3 (SD 14) years with a predominance of men. Almost 10% developed severe dengue (plasma leakage 67%, clinical bleeding 25%, and organ involvement 25%). Fever, headache, retro-orbital pain, diarrhea, and warning signs such as abdominal pain, clinical fluid accumulation, and persistent vomiting were the most common clinical presentations. Thrombocytopenia, leukopenia, elevated HCT levels, and ALT/AST were common laboratory findings.

**Conclusions:**

Severe dengue was mostly attributable to plasma leakage with warning signs, especially abdominal pain, clinical fluid accumulation, persistent vomiting, and altered hematological parameters which might assist in the early prediction of severe dengue.

## 1. Background

Dengue is an arthropod-borne viral disease caused by the dengue virus (DENV), a single-stranded RNA virus of the genus *Flavivirus* that is transmitted to humans by infected mosquitoes of the species *Aedes aegypti* and sometimes *Aedes albopictus* [1]. DENV may have four serotypes (DEN 1–4). The cocirculation of all these serotypes during epidemics increases the risk of infecting an individual as many as four times with different serotypes, resulting in more severe disease [1, 2]. Dengue infection may present with a wide spectrum of clinical manifestations, ranging from conventional influenza symptoms to very severe bleeding or plasma leakage, resulting in dengue shock syndrome [3].

Billions of people living in the tropical climate of Southeast Asia, sub-Saharan Africa, and Latin America are vulnerable to dengue infection [2, 4]. Almost 390 million people worldwide are infected by the dengue virus annually, resulting in almost 20,000 deaths [5, 6]. This high burden of disease and mortality has made dengue a major public health concern for these countries. Rapid urbanization with minimal and unplanned sanitation facilities, availability of fertile breeding spaces for mosquitoes, lack of adequate vector control, and climatic changes have accelerated the widespread dengue infection in these countries [7].

Bangladesh is considered one of the endemic zones for dengue in the southeast Asian region. Historically, the country faced a dengue epidemic in 2000 for the first time, with a reported 5,521 confirmed dengue cases and 92 deaths [8]. Since then, several incidences of dengue outbreaks have occurred in this country, with a reported number of almost 32,000 dengue cases in the last two decades [9]. However, during the epidemic that occurred in 2019, all previous records exceeded 100,000 confirmed cases and 164 deaths from all over the country [10, 11].

All four serotypes of DENV were reported circulating in Bangladesh during different outbreaks. During the initial outbreaks of 2002, mostly DEN-3 was isolated, while DEN-1 and DEN-2 were the most commonly circulating strains in the outbreaks between 2013 and 2016 [12, 13]. However, re-emergence of the DEN-3 serotype was reported during recent epidemics [14]. This transition of DENV serotypes across the outbreaks and potential reinfection may result in a temporal change in the clinical manifestations of dengue infection. Hemorrhagic manifestations (skin rash, melena, gum bleeding, epistaxis, conjunctival hemorrhage, etc.) were more common in previous epidemics, while increasing trends in GIT symptoms (e.g., anorexia, abdominal pain, and diarrhea) as well as evidence of plasma leakage (e.g., pleural effusion, ascites, and shock) were observed in recent epidemics [9].

The management of a large number of dengue patients during an outbreak is a major challenge for a resource-poor health system in a country such as Bangladesh. The WHO suggested a new dengue classification system in 2009 for adequate clinical management of dengue cases [15]. It is important to identify and triage high-risk cases of severe dengue early to prioritize their management. However, the changing pattern in the clinical presentation of dengue during recent epidemics has made this issue more challenging. For prompt diagnosis and adequate management, thorough knowledge of the clinical presentation of dengue cases from ongoing or recent outbreaks plays a crucial role. Hence, the present study aimed to investigate the epidemiological, clinical, and laboratory profiles as well as the disease severity of the dengue cases from the outbreak that occurred in Bangladesh during 2019.

## 2. Methods

### 2.1. Study Design and Setting

The present one was a cross-sectional descriptive study conducted in Dhaka Medical College Hospital and Dr Sirajul Islam Medical College Hospital, Dhaka, two tertiary care hospitals situated in Dhaka, Bangladesh, between June and September 2019. Before commencing the study, the study protocol was approved by the Ethical Review Committee of Dhaka Medical College (memo no. MEU-DMC/ECC/2019/251).

### 2.2. Study Participants

All the consecutive adult patients (aged more than 18 years) admitted to the hospital with a confirmed dengue infection were included in the present study. The inclusion criteria were as follows: clinically and serologically confirmed dengue infection evidenced by a positive dengue NS1 antigen and/or IgM antibody, age ≥18 years of either sex. Before admission, all the clinically suspected dengue patients were tested for specific dengue NS1 antigen if they presented within the sixth day of onset of symptoms or IgM antibody if they presented after the sixth day of onset of symptoms. Enzyme-linked immunosorbent assays (ELISA) using the DENV Detect™ NS1 ELISA Kit and the DENV Detect™ IgM/IgG ELISA Kit (InBios International, Inc., USA) was done for detection of dengue NS1 antigen, IgM, and IgG, respectively. The exclusion criteria were as follows: pediatric patients, pregnancy, and mixed infection (coinfection by other organisms such as chikungunya virus or plasmodium along with DENV).

### 2.3. Data Collection

Patients fulfilling the inclusion criteria were recruited for the study after they provided an informed written consent. Patients' sociodemographic and disease-related information, such as clinical presentations and laboratory parameters, were recorded from patients in a case-record form. A face-to-face interview and thorough clinical examinations were performed by attending physicians on admission to investigate the clinical presentations. Patients' comorbidities were reported based on their medical records or according to their statements if records were not available. The severity of dengue infection was defined on the date of discharge according to the WHO's 2009 classification as nonsevere (with or without warning signs) and severe dengue. All dengue patients received standard management according to the WHO recommendations [16].

### 2.4. Laboratory Investigations

Laboratory investigations including a complete blood count (using the Sysmex XN-2000™ Hematology Autoanalyzer, Sysmex Corporation), serum alanine aminotransferase (ALT), aspartate aminotransferase (AST), and creatinine were performed on patient admission, and a complete blood count was repeated every day to track hematocrit and platelet count changes.

The definition of thrombocytopenia was a platelet count of 100,000 platelets/*μ*L or less; leukopenia was a white blood cell count 5000 cells/*μ*L, a raised hematocrit was >48%, or ≥20% increase than the baseline, a raised ALT/AST level was >50 IU/L; and a raised creatinine level was >1.2 mg/dl [11, 17].

### 2.5. Case Definitions

We have used the WHO 2009 criteria for defining dengue cases [16]. Confirmed dengue infection was defined as “history of acute fever with at least two of the following symptoms: headache, retro-orbital or ocular pain, myalgia, arthralgia, rash, a positive tourniquet test (defined as the presence of >20 petechiae per 1 square inch), or leukopenia (defined as a white blood cell count <5000 cells/mm^3^); and being positive for NS1 antigen or IgM antibody.” Nonsevere dengue was defined as “having acute fever with at least two of the following criteria: headache, myalgia, arthralgia, nausea, vomiting, rash, a positive tourniquet test, or leukopenia.” Warning signs were defined as the “presence of abdominal pain or tenderness, persistent vomiting (vomiting with signs of dehydration), clinical fluid accumulation, bleeding from mucosal areas including the nose, gums, gastrointestinal tract or vagina, lethargy, restlessness, liver enlargement >2 cm, and increase in hematocrit (≥20%) concurrent with rapid decrease in platelet count (<100000/*μ*L).” Severe dengue was defined as “having severe plasma leakage (defined as plasma leakage with shock or respiratory distress (respiratory rate <24 breaths/min with oxygen saturation <95% in room air and/or requiring oxygen therapy)), severe clinical bleeding (spontaneous bleeding from mucosal areas that necessitated a blood transfusion), or severe organ involvement (liver impairment (AST >1000 IU/L and/or ALT >1000 IU/L), renal impairment (serum creatinine >2 times above baseline), myocarditis, and/or encephalitis)” [16]. Clinical fluid accumulation included pleural effusion (diagnosed as homogenous density in the dependent portion in the chest X-ray) and ascites (seen as an anechoic space on ultrasound of the whole abdomen) [18].

### 2.6. Statistical Analysis

Descriptive statistics were used to represent the findings. For the normally distributed continuous variables, the mean with standard deviation (SD) was used, and for the non-normally distributed continuous variables, medians with interquartile range (IQR) were used. For categorical variables, a frequency distribution with percentage was used. All statistical analyses were done in STATA version 17.0.

## 3. Results

### 3.1. Sociodemographic Characteristics

The average age of the patients was 33.3 years (SD 14 years), and almost half of them were young adults in the age group of 18 to 30 years. Almost 60% of the patients were male, and 76% belonged to middle- or high-income families (Table 1).

### 3.2. Severity of Dengue

**Figure 1:**
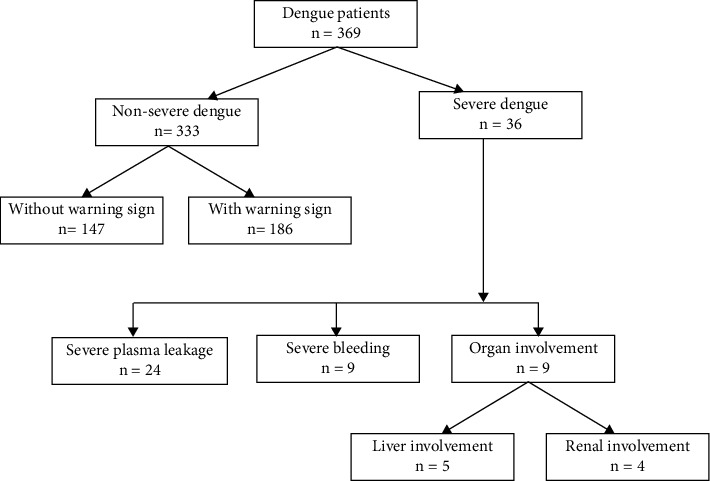
Severity of disease among the dengue patients (*n* = 369).

A total of 369 patients suffering from confirmed dengue infection were enrolled in the present study. Among them, a total of 333 patients (90.2%) suffered from nonsevere dengue (44%, *n* = 147 without warning signs, and 56%, *n* = 186 with warning signs). Another 36 patients (9.8%) were suffering from severe dengue. Among severe dengue patients, severe plasma leakage was the most common manifestation (*n* = 24, 66.7), followed by severe clinical bleeding (*n* = 9, 25%) and organ involvement (liver involvement in 5 patients and renal involvement in 4 patients) (Figure 1).

### 3.3. Clinical Presentations

All the dengue patients included in the present study presented with fever during their admission. Headache and nausea/vomiting were also common manifestations in dengue patients and were present in 60% and 67% of patients, respectively. Other commonly presenting symptoms were anorexia (44%), retro-orbital pain (36%), diarrhea (36%), back pain (28%), myalgia (20%), arthralgia (20%), etc. Abdominal pain was the most common warning sign (37%), followed by clinical fluid accumulation (pleural effusion/ascites) (22.5%) and persistent vomiting (16.5%). Among bleeding manifestations, gum bleeding (6%) and melena (5%) were the most common, followed by menorrhagia (5%), hematuria (3%), and epistaxis (2%). Almost 13.5% of patients presented with pleural effusion, 9% presented with ascites, and 4% presented with shock (Table 2).

### 3.4. Laboratory Profile

A total of 90% of patients were confirmed for dengue infection by a positive NS1 antigen test, and the remaining 10% were confirmed by a positive IgM antibody test. Almost 7% of patients (*n* = 25) tested positive for IgG antibodies. Almost 18% of patients presented with raised HCT (median 40.4 and IQR 6.6). Thrombocytopenia and leukopenia were present in more than half of the patients (58% and 51%, respectively). Raised ALT was present in 42% of patients, and raised AST was present in 31% of patients. Renal impairment was observed in almost 8.7% of patients (Table 3).

## 4. Discussion

Bangladesh has faced a frequent dengue epidemic during the last two decades since the first epidemic occurred in 2000. It has been reported that all four serotypes of DENV circulate alternately during different outbreaks. For example, during the initial outbreaks that occurred in 2000 and 2002, DEN-3 was the predominant strain, which re-emerged during the recent epidemics that occurred in 2018 and 2019 after a lapse in 2013 and 2016, when DEN-1 and DEN-2 were the predominant strains [12–14]. This changing pattern in circulating serotypes may impact the clinical manifestations and disease severity of dengue fever in patients, as an individual might potentially be infected by all four serotypes, and subsequent infections increase the severity of the disease.

Among our 369 included dengue patients, a total of 33 (10%) developed severe dengue according to the case definition of the WHO 2009 criteria. A previous study conducted in pediatric dengue cases from the 2019 epidemic reported that almost 28% of children developed severe dengue, which is much higher than our finding [19]. However, being a child itself is evidenced as a risk factor for developing severe dengue, which might explain this finding [20]. The proportion of patients with severe dengue in our study was slightly higher than that in a previous study conducted during the same epidemic in a nonendemic zone of the country where almost 6% of patients developed severe dengue [21]. As the present study was conducted in a facility in Dhaka, which is considered endemic for dengue in the country, the majority of the cases in previous epidemics were reported from this region, and the seroprevalence of DENV is high in this part of the country [13], it is possible that a large number of secondary cases were present in the patient cohort. Almost 10% of our patients tested positive for IgG, which indicated a secondary infection. Subsequent infection by different dengue strains increases disease severity [20]. Among our patient cohort, plasma leakage was the most common form of severe dengue, accounting for more than one-third of all severe dengue cases. The same manifestation was observed in another cohort of patients from the same outbreak, where 68% of severe cases were attributable to severe plasma leakage [21]. Although dengue hemorrhagic fever was more common during initial outbreaks, a declining trend in this case was observed in recent epidemics where severe plasma leakage replaced hemorrhage [9]. Plasma leakage was also commonly reported manifestation of severe dengue from a recent outbreak in Singapore [22]. The re-emergence of the DEN-3 serotype, which mainly induces severe plasma leakage, might contribute to this clinical shift. Organ involvement was also common in patients with severe dengue included in our study as well as in previous studies conducted during the same epidemic [9, 11, 21]. Severe hepatic impairment and renal impairment were common manifestations of organ involvement in severe dengue.

Fever was present in all the dengue patients on admission, as most of them were admitted to the hospital during the febrile phase of the disease. Among other clinical manifestations, headache, nausea/vomiting, retro-orbital pain, myalgia, arthralgia, and gastrointestinal symptoms such as diarrhea and constipation were also common among our patients. These symptoms were also frequently reported in other contemporary studies [11, 21]. A gradual increase in gastrointestinal symptoms was reported in recent epidemics, which is also supported by our findings [9]. Almost half of the dengue patients presented with one or more warning signs. Among those, abdominal pain, clinical fluid accumulation (pleural effusion/ascites), and persistent vomiting were most common. These warning signs were also commonly reported in other studies conducted during the epidemic [11, 21]. Among hemorrhagic features, gum bleeding and melena were commonly reported in our patients, which comply with the findings of some other studies [11, 21]. However, the prevalence of hemorrhagic manifestations was less common compared to previous epidemics, while evidence of plasma leakage was more common [9].

Among the hematological parameters, thrombocytopenia, and leukopenia were the most common findings present in almost half of the patients. Although the prevalence of thrombocytopenia found in our study was similar to a previous epidemic that occurred in Bangladesh during 2002 [23], it was higher compared to a study conducted in a different hotspot of the country during the same epidemic [21]. Raised hematocrit levels, which are one of the cardinal features of plasma leakage, were found in 18% of patients, which conforms to the findings from other studies [11, 21]. Evidence of organ involvement was also commonly present in our study patients. Raised serum ALT and/or AST levels were present in almost half of the patients, and raised serum creatinine levels were present in almost 8% of patients. The prevalence of increased liver enzymes is similar to the reports of other studies conducted in the same region [9]. Although it was higher compared to a study conducted in a nonendemic zone of the country [21]. Although the values of these parameters did not reach the level of severe dengue in the majority of the cases, this early rise in liver-specific enzymes may play a role in the prediction of severe dengue.

The present study highlights the epidemiological, clinical, and laboratory characteristics of dengue patients infected during the 2019 epidemic in Dhaka, Bangladesh. However, the study has several limitations. First, in this observational study, we only included hospital-admitted patients which might skip a number of nonsevere cases that did not require admission. Second, confirmation of the dengue serotype was not performed in the present study. Third, differentiation of primary and secondary dengue infections was not performed.

## 5. Conclusions

A substantial number of patients developed severe dengue during the 2019 epidemic in Bangladesh, and the majority of their symptoms manifested as plasma leakage rather than dengue hemorrhagic fever. Conventional clinical features such as fever, headache, myalgia, and rash, along with gastrointestinal discomfort, were common in dengue patients. Warning signs, such as abdominal pain and persistent vomiting, as well as evidence of organ involvement, such as increased liver enzymes, were also common. Clinicians should consider these findings when approaching a febrile patient, especially during an epidemic period.

## Figures and Tables

**Table 1:** Sociodemographic characteristics of the dengue patients (*n* = 369).

Characteristics	*n*	%
Age (years)	Mean = 33.27	SD = 14.09
18–30	205	55.56
31–40	85	23.04
41–50	27	7.32
51–60	27	7.32
>60	25	6.78
Sex		
Male	221	59.89
Female	148	40.11
Educational attainment		
Primary	18	13.14
Secondary	22	16.06
Higher secondary	49	35.77
University graduate	48	35.04
Monthly family income		
Low (<BDT 20,000)	88	23.92
Middle (BDT 20,000 to 40,000)	147	39.86
High (>BDT 40,000)	134	36.22
Comorbidities		
Diabetes mellitus	30	8.13
Hypertension	25	6.78
Cardiovascular disease	10	2.71

BDT, Bangladeshi taka.

**Table 2:** Clinical presentation of the dengue patients (*n* = 369).

Clinical presentation^∗^	*n*	%
Fever	369	100.00
Headache	223	60.43
Retro-orbital pain	132	35.77
Back pain	104	28.18
Myalgia	73	19.78
Sore throat	49	13.28
Generalized weakness	112	30.35
Rash	64	17.34
Arthralgia	75	20.33
Nausea/vomiting	249	67.48
Dehydration	89	24.12
Diarrhea	134	36.31
Anorexia	162	43.90
Constipation	32	8.67
Warning signs		
Abdominal pain	136	36.86
Persistent vomiting	61	16.53
Clinical fluid accumulation (pleural effusion/ascites)	83	22.49
Hepatomegaly	10	2.71
Splenomegaly	5	1.36
Bleeding manifestation		
Gum bleeding	22	5.96
Epistaxis	7	1.90
Hematuria	12	3.25
Melena	19	5.15
Menorrhagia	18	4.88
Positive tourniquet test	13	3.52
Evidence of plasma leakage		
Pleural effusion	50	13.55
Ascites	33	8.94
Shock	17	4.61

^∗^Multiple response considered.

**Table 3:** Laboratory parameters of the dengue patients (*n* = 369).

Continuous data	Median	IQR
HCT (%)	40.4	6.6
Hemoglobin (g/dl)	13.4	2.4
Platelet (cells × 10^3^ cells/*μ*L)	70.0	115
WBC (cells × 10^3^ cells/*μ*L)	4.2	3.4
Bilirubin (mg/dl)	1.2	1.58
ALT	110.0	12
AST	78.0	118
Serum creatinine	1.0	0.4

Categorical data	*n*	%

NS1		
Positive	330	89.43
Negative	39	10.57
IgM		
Positive	39	10.57
Negative	330	89.43
IgG		
Positive	25	6.78
Negative	344	93.22
HCT (%)		
≤48	303	82.11
>48	66	17.89
Hemoglobin (g/dl)		
≥12	306	82.93
<12	63	17.07
Platelet (cells × 10^3^ cells/*μ*L)		
≥100	154	41.73
<100	215	58.27
WBC (cells × 10^3^ cells/*μ*L)		
≥5	180	48.78
<5	189	51.22
Bilirubin (mg/dl)		
<2	363	98.37
≥2	6	1.63
ALT (IU/l)		
<50	214	57.99
≥50	155	42.01
AST (IU/l)		
<50	253	68.56
≥50	116	31.44
Serum creatinine (mg/dl)		
<1.2	337	91.33
≥1.2	32	8.67

HCT, hematocrit; WBC, white blood cell; NS1 Ag, nonstructural antigen-1; ALT, alanine aminotransferase; AST, aspartate aminotransferase.

## Data Availability

Patients' data will be available upon reasonable request from the corresponding author.
